# Comparative analysis of corneal parameters in simple myopic anisometropia using Scheimpflug technology

**DOI:** 10.3389/fbioe.2024.1366408

**Published:** 2024-05-22

**Authors:** Di Wang, Yue Chang, Weijin Nan, Yan Zhang

**Affiliations:** ^1^ Department of Ophthalmology, The Second Hospital of Jilin University, Changchun, China; ^2^ Department of Ophthalmology, Shanghai General Hospital, Shanghai Jiao Tong University School of Medicine, Shanghai, China

**Keywords:** corneal biomechanics, corneal topography, anisometropia, myopia, Scheimpflug

## Abstract

**Purpose:**

This study aims to investigate the differences in binocular corneal parameters and their interrelation with binocular biometric parameters asymmetry in patients with simple myopic anisometropia, thereby elucidating the influence of myopia process on various corneal parameters.

**Methods:**

In this cross-sectional study, 65 patients with anisometropia in monocular myopia were included. They were divided into low anisometropia group: 3.00D<Δ spherical equivalent (SE)≤−1.00D (Δ represents the difference between the two eyes, i.e., myopic data minus emmetropic data) and high anisometropia group: ΔSE ≤ −3.00D. Corneal and ocular biometric parameters were measured using Pentacam, Corvis ST, and IOL Master 700. Statistical analyses focused on the binocular corneal parameters asymmetry, using the contralateral emmetropia as a control.

**Results:**

The mean age of participants was 18.5 ± 1.3 years, with the average SE for myopia and emmetropia being −2.93 ± 1.09D and −0.16 ± 0.41D, respectively. The central corneal thickness (CCT), flat keratometry (Kf), keratometry astigmatism (Ka), total corneal aberration (6 mm) (TOA), surface variance index (ISV), vertical asymmetry index (IVA), stress-strain index (SSI), and first applanation stiffness parameter (SPA1) and ambrosia relational thickness-horizontal (ARTh) showed significant differences between anisometropic fellow eyes (*p* < 0.05). There were significant differences in ΔIVA, Δ the difference between the mean refractive power of the inferior and superior corneas (I-S), Δ deviation value of Belin/Ambrósio enhanced ectasia display (BAD-D), Δ deformation amplitude ratio max (2 mm) (DAR)and Δ tomographic biomechanical index (TBI) (*p* < 0.05) in two groups. Asymmetry of corneal parameters was correlated with asymmetry of ocular biometric parameters. Anisometropia (ΔSE) was positively correlated with ΔIVA (r = 0.255, *p* = 0.040), ΔBAD-D (r = 0.360, *p* = 0.006), and ΔSSI (r = 0.276, *p* = 0.039) and negatively correlated with ΔDAR (r = −0.329, *p* = 0.013) in multiple regression analysis. Δ mean keratometry (Km), Δ anterior chamber depth (ACD), and Δ biomechanically corrected intraocular pressure (bIOP) were also associated with binocular corneal differences.

**Conclusion:**

Compared to contralateral emmetropia, myopic eyes have thinner corneas and smaller corneal astigmatism. Myopic corneas exhibit relatively more regular surface morphology but are more susceptible to deformation and possess marginally inferior biomechanical properties. In addition, there is a certain correlation between anisometropia and corneal parameter asymmetry, which would be instrumental in predicting the development of myopia.

## 1 Introduction

The cornea, a biological tissue enriched with extracellular matrix, functions as a principal refractive medium and constitutes the outermost fibrous structure of the human eye. It is responsible for nearly two-thirds of the ocular refractive power and plays a pivotal role in safeguarding the intraocular tissues. These critical functions are contingent upon the cornea’s geometric configuration and its biomechanical attributes. Notably, variations in corneal parameters are closely related to myopia (Yu et al., 2020; Ma et al., 2021; Sedaghat et al., 2021). On the one hand, axial elongation of the eye leads to the development of myopia. Research has elucidated that these structural alterations predominantly arise from dysregulation in collagen metabolism within the sclera, culminating in scleral thinning and a reduction in mechanical properties (Markov et al., 2018). Given that both the cornea and sclera form integral components of the eyeball’s outer wall, featuring analogous arrangements of collagen fiber layers, the cornea can serve as an indirect indicator of scleral parameters that are otherwise challenging to quantify (Nguyen et al., 2020), helping to elucidate the pathogenesis and progression of myopia. On the other hand, the cornea is the primary focus in refractive correction. Procedures modify the refractive power through selective removal of corneal tissue, whereas orthokeratology lenses induce alterations in refractive status by reshaping the cornea, both leading to changes in its morphological and biomechanical parameters (Guo et al., 2019). Therefore, an enhanced understanding of the multi-dimensional corneal parameters will help deepen and broaden the research on myopia.

Current research, through comparative analyses across distinct individuals (Yu et al., 2020; Ma et al., 2021; Sedaghat et al., 2021), has substantiated the variability of corneal parameters across varying degrees of myopia. Nonetheless, *in vivo* measurements of these parameters are subject to an array of confounding factors. While it is feasible to adjust for elements like age, biomechanically corrected intraocular pressure (bIOP), and central corneal thickness (CCT), the mitigation of individual disparities such as hormonal levels and corneal hydration remains challenging, and recent studies have found that estrogen levels can affect the refractive status and biomechanical properties of the cornea (Kling and Hafezi, 2017; Leshno et al., 2020). Consequently, this leads to variances in research outcomes, and the correlation between alterations in corneal parameters and myopia remains ambiguous. Anisometropic patients, presenting differing refractive states within the same individual, offer a unique advantage in minimizing the impact of these confounding factors. Furthermore, the subjects with simple myopic anisometropia in this study exhibit a natural dichotomy between myopic and emmetropic states, rendering them exemplary for investigating the interplay between myopia and corneal transformations. This approach enables a more profound exploration of the shifts in corneal morphology and biomechanics in myopic process (spherical equivalent (SE) decreased or axial length (AL) increased).

## 2 Materials and methods

### 2.1 Study population

This investigation adhered to the ethical guidelines delineated in the Helsinki Declaration and received approval from the Ethics Committee of the Second Hospital of Jilin University (Approval No. 205 of 2023 Research Review). Employing a cross-sectional design, the study comprised 65 patients (130 eyes) diagnosed with simple myopic anisometropia, who sought medical attention at our institution between March 2022 and June 2023. Prior to inclusion, all participants provided their informed consent, duly documented and verified.

Eligibility for inclusion in the study of simple myopic anisometropia required specific criteria: post-ciliary muscle paralysis (using compound tropicamide eye drops), a degree in myopic eye of sphere (DS) ≤ −0.50D, an emmetropic eye range of −0.50D ≤ DS ≤ 0.75D, ΔSE ≤ −1.00D and −1.00D ≤ Δcylinder (DC) ≤ 1.00D (Δ represents the difference between the two eyes, i.e., myopic data minus emmetropic data). Subjects were ineligible if they met any of the following criteria: 1) Existence of corneal or ocular diseases (e.g., keratoconus, corneal ulcer, corneal dilation, glaucoma); 2) History of ocular surgery or trauma; 3) Manifest strabismus or amblyopia; 4) Systemic diseases influencing corneal parameters; 5) Current pregnancy or menstrual phase; 6) Recent use of contact lenses: cessation period of 1 week for soft lenses, 1 month for rigid gas permeable lenses, and 3 months for Orthokeratology lenses. Participants were further categorized into two subgroups based on the degree of anisometropia: 1) Low anisometropia group (−3.00D<ΔSE ≤ −1.00D); 2) High anisometropia group (ΔSE ≤ −3.00D).

### 2.2 Methods

Participants underwent comprehensive ophthalmological assessments, encompassing tests for visual acuity, computerized and subjective optometry, along with slit lamp and fundus examinations. AL, vitreous cavity length (VL), anterior chamber depth (ACD), and lens thickness (LT) were quantified using the IOL Master 700 (Carl Zeiss Meditec, Jena, Germany).

Corneal topography and biomechanical properties were evaluated employing a Pentacam in conjunction with the Corvis ST (Oculus, Wetzlar, Germany). A Scheimpflug camera was utilized to capture detailed images of the anterior segment of the eye, while ultra-high-speed Scheimpflug tomography facilitated the acquisition of dynamic corneal images under jet pulse stimulation. All measurements were conducted in a controlled, darkened environment by certified technicians. To ensure accuracy, each examination was repeated thrice, and the mean value was recorded. Only measurements with a quality index of “OK” were considered valid for inclusion in the subsequent analysis.

### 2.3 Statistical analysis

Statistical analyses were executed using SPSS software (IBM SPSS Statistics for Windows, Version 26.0, IBM, Armonk, New York, United States). To assess data normality, the Kolmogorov-Smirnov test was employed. Variables adhering to a normal distribution were expressed as mean ± standard deviation, otherwise depicted as median (P25, P75). A self-controlled observational approach was adopted for the myopic and emmetropic eyes of the study participants, enabling the acquisition of differential binocular data, which was then analyzed using either the Paired-Sample *t*-Test or the Wilcoxon matched-pairs signed rank test. The disparity in corneal parameters across distinct anisometropic groups was examined using the Independent-Sample *t*-Test or the Wilcoxon Signed Rank Test. Correlations between corneal parameters and ocular biometric parameters were investigated utilizing either Pearson’s correlation coefficient or Spearman’s rank correlation coefficient. Multiple linear regression with a stepwise approach was applied to elucidate the relationship between corneal and ocular biological parameters. A significance threshold was established at *p* < 0.05.

## 3 Results

The study encompassed 65 patients (130 eyes) diagnosed with simple myopic anisometropia, presenting an average age of 18.5 ± 1.3 years (range: 17–23 years). Of these participants, 41 were categorized into the low anisometropia group, while 24 were classified in the high anisometropia group.

### 3.1 Comparison of ocular biometric and corneal parameters in anisometropic fellow eyes

**FIGURE 1:**
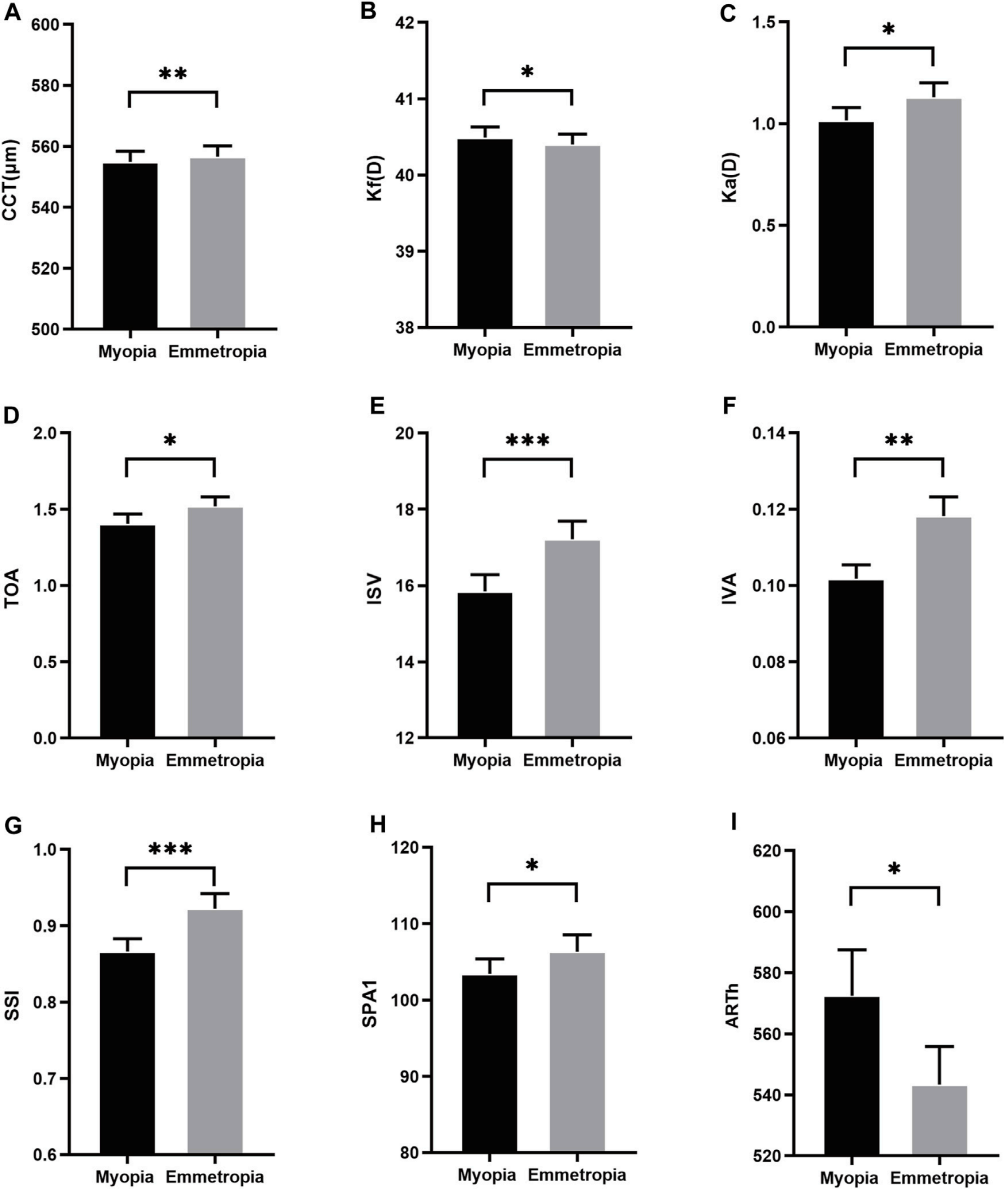
Histogram comparison of CCT, Kf, Ka, TOA, IVA, SSI, SPA1 and ARTh **(A–I)** in anisometropia fellow eyes. *means *p* < 0.05, **means *p* < 0.01, ***means *p* < 0.001.

In this cohort, the mean SE and AL in myopia were −2.93 ± 1.09D and 25.44 ± 0.92 mm, respectively, while in emmetropia, these values were recorded as −0.16 ± 0.41D and 24.24 ± 0.65 mm. As depicted in Table 1, a comparative assessment of biological parameters revealed significant disparities in AL, VL, ACD, and LT (*p* < 0.05). It was observed that the CCT and keratometry astigmatism (Ka) in myopia were considerably lower compared to emmetropia, whereas the flat keratometry (Kf) was higher (*p* < 0.05). Regarding corneal morphological parameters, notable differences were discerned in total aberrations (6 mm) (TOA), index of surface variance (ISV), and index of vertical asymmetry (IVA) between myopic and emmetropic eyes (*p* < 0.05). Furthermore, in the biomechanical parameters analysis, significant distinctions were found in the stress-strain index (SSI), the first applanation stiffness parameter (SPA1), and the ambrosia relational thickness-horizontal (ARTh) when comparing myopic eyes to their emmetropic counterparts (*p* < 0.05) (Figures 1A–I).

**TABLE 1:** Comparison of ocular biometric and corneal parameters in anisometropic fellow eyes.

	Myopia eye	Emmetropia eye	D-value 95%CI	*t*/Z	*p*-value
Ocular biometric parameters					
SE	−2.93 ± 1.09	−0.16 ± 0.41	−2.77 (−3.05, −2.50)	−20.183	**<0.001**
DS	−2.59 ± 1.01	0.25 ± 0.38	−2.84 (−3.11, −2.58)	−21.575	**<0.001**
DC	−0.67 ± 0.53	−0.82 ± 0.52	0.14 (0.03,0.25)	2.546	**0.013**
bIOP	15.55 ± 2.75	15.58 ± 3.50	−0.04 (−0.60,0.53)	−0.126	0.900
AL	25.44 ± 0.92	24.24 ± 0.65	1.20 (1.06,1.33)	18.018	**<0.001**
VL	18.17 ± 0.89	17.01 ± 0.64	1.16 (1.03,1.29)	17.699	**<0.001**
ACD	3.28 ± 0.24	3.21 ± 0.25	0.06 (0.05,0.08)	7.631	**<0.001**
LT	3.43 ± 0.18	3.46 ± 0.21	−0.02 (−0.04, −0.01)	−3.066	**0.003**
Corneal biometric parameters					
CCT	554.89 ± 27.96	556.63 ± 28.29	−1.74 (−2.93, −0.55)	−2.914	**0.005**
CV	60.81 ± 4.32	60.87 ± 4.55	−0.06 (−0.19, −0.08)	−0.836	0.406
CEC	3006.25 ± 240.84	3014.57 ± 244.17	−8.32 (−29.03,12.38)	−0.803	0.425
Kf	40.49 ± 1.12	40.40 ± 1.09	0.09 (0.01,0.17)	2.256	**0.027**
Ks	41.51 ± 1.12	41.54 ± 1.11	−0.02 (−0.12,0.07)	−0.527	0.600
Km	41.00 ± 1.09	40.98 ± 1.06	0.02 (−0.05,0.09)	0.703	0.485
Ka	−1.02 ± 0.52	−1.13 ± 0.56	−0.12 (−0.22,−0.01)	−2.283	**0.026**
Corneal morphological parameters					
Q	−0.32 (−0.41, −0.25)	−0.31 (−0.43, −0.25)	0.01 (−0.01, 0.03)	−0.442	0.658
PCE	2.06 ± 2.37	1.83 ± 2.47	0.23 (−0.32,0.78)	0.838	0.405
TOA	1.41 ± 0.50	1.52 ± 0.50	−0.11 (−0.21, −0.01)	−2.268	**0.027**
HOA	0.36 ± 0.11	0.37 ± 0.10	−0.01 (−0.04, 0.01)	−1.167	0.247
ISV	15.85 ± 3.53	17.22 ± 3.81	−1.37 (−2.02, −0.71)	−4.176	**<0.001**
IVA	0.10 ± 0.03	0.12 ± 0.04	−0.02 (−0.03, −0.01)	−3.535	**0.001**
IHA	4.11 ± 3.14	4.22 ± 3.54	−0.10 (−1.03,0.82)	−0.226	0.822
I-S	0.15 ± 0.38	0.16 ± 0.43	−0.01 (−0.10,0.07)	−0.252	0.802
BAD-D	0.91 ± 0.46	0.98 ± 0.54	−0.07 (−0.15,0.01)	−1.806	0.076
Corneal biomechanical parameters					
SSI	0.87 ± 0.12	0.92 ± 0.15	−0.06 (−0.08,−0.03)	−4.200	**<0.001**
SPA1	103.48 ± 14.32	106.34 ± 16.49	−2.86 (−5.67,−0.05)	−2.040	**0.046**
ARTh	572.46 ± 112.89	543.36 ± 93.13	29.11 (6.69,51.53)	2.602	**0.012**
DAR	4.30 ± 0.43	4.24 ± 0.41	0.06 (0.00,0.12)	1.942	0.057
IR	8.81 ± 1.16	8.68 ± 1.33	0.13 (−0.09,0.35)	1.210	0.232
CBI	0.00 (0.00, 0.01)	0.00 (0.00, 0.03)	0.01 (−0.02,0.05)	−0.807	0.420
TBI	0.18 ± 0.15	0.21 ± 0.18	−0.03 (−0.07, 0.02)	−1.240	0.220

Bold values indicate *p* < 0.05.

### 3.2 Comparison of asymmetry in corneal and ocular biometric parameters across low and high anisometropic groups

**FIGURE 2:**
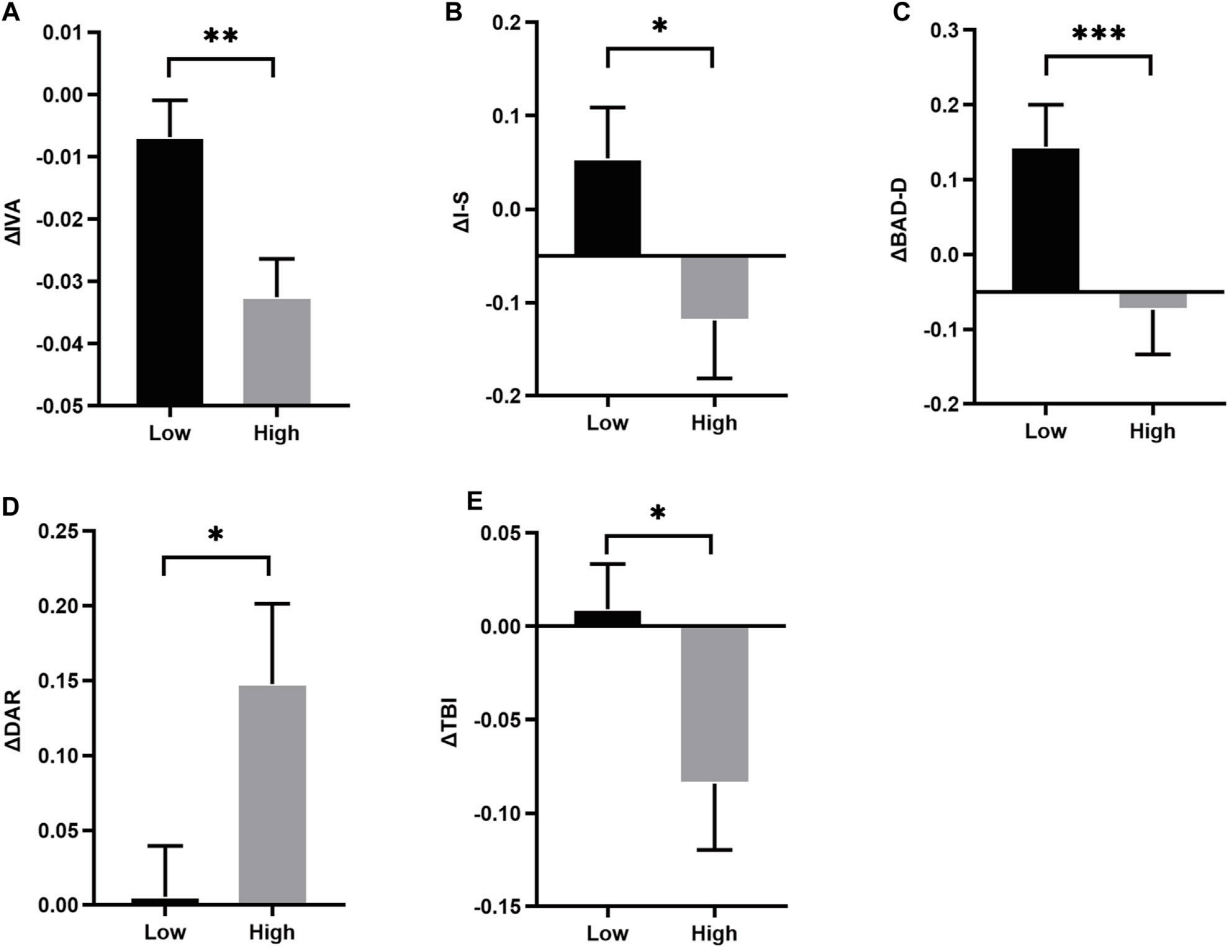
Histrogram comparison of ΔIVA, ΔI-S, ΔBAD-D, ΔDAR and ΔTBI **(A–E)** in anisometropia fellow eyes. *means *p* < 0.05, **means *p* < 0.01, ***means *p* < 0.001.

The overall anisometropia averaged at −2.77 ± 1.11D (range: −1.00∼−5.50D), with ΔSE of −2.09 ± 0.58D and −3.94 ± 0.76D in low and high anisometropic groups, respectively. There was no difference in age and gender between the two groups (*p* > 0.05), and the difference in binocular refractive status and biological parameters were significantly different, including ΔSE, ΔDS, ΔAL, ΔVL (*p* < 0.05). Asymmetry of corneal parameters, ΔIVA, Δ difference between the mean refractive power of the inferior and superior corneas (I-S), Δ deviation value of Belin/Ambrósio enhanced ectasia display (BAD-D), Δ deformation amplitude ratio max (2 mm) (DAR), Δ tomographic biomechanical index (TBI) were statistically significant in both groups (*p* < 0.05) (Figures 2A–E). More detailed data can be found in the Supplementary Material.

### 3.3 Correlation of asymmetry between corneal parameters and ocular biometric parameters

**FIGURE 3:**
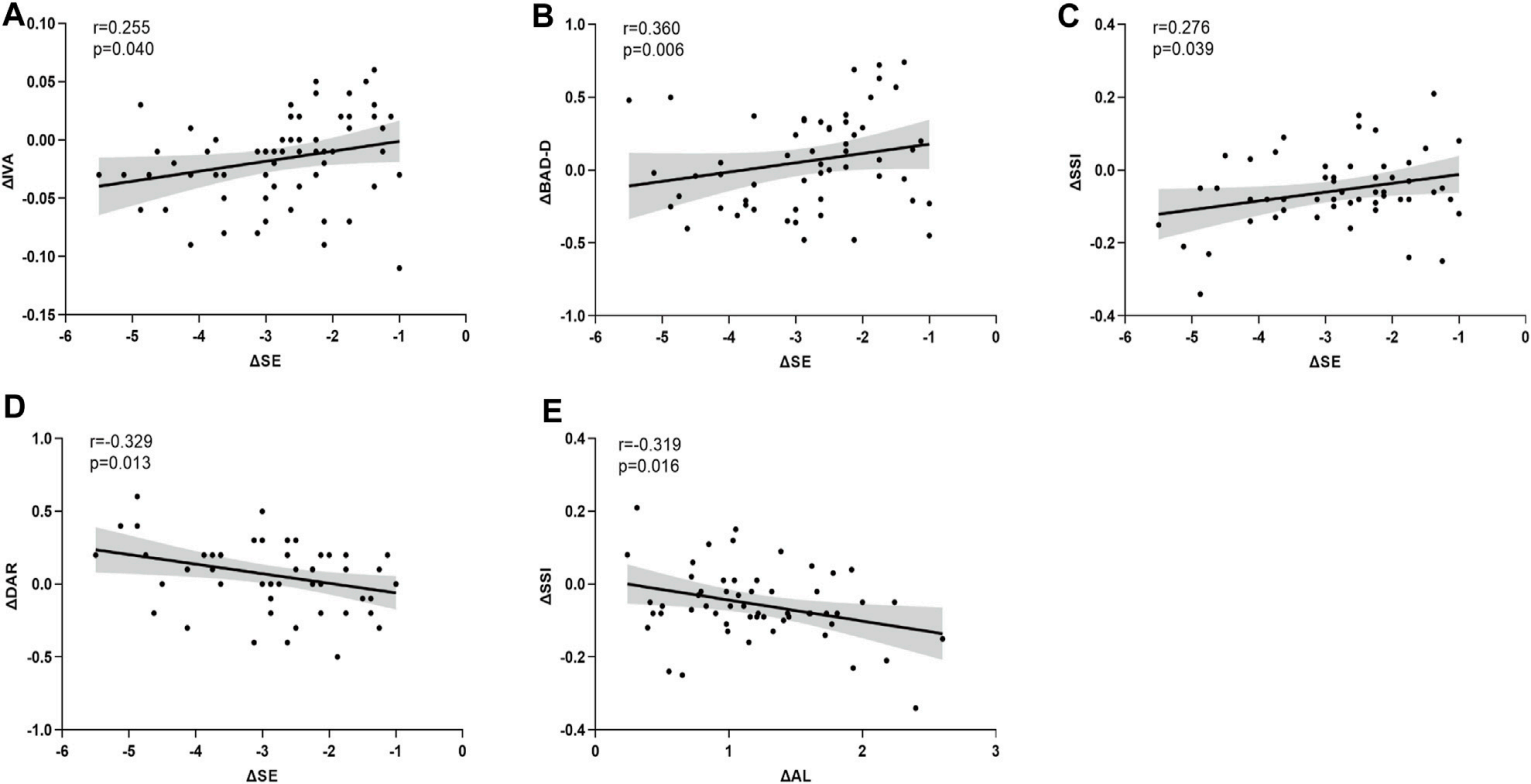
The asymmetry of corneal parameters are significantly correlated with anisometropia **(A–E)**. SE is significantly correlated with ΔIVA **(A)**, ΔBAD-D **(B)**, ΔSSI **(C)**, ΔDAR **(D)**, and ΔAL is significantly correlated with ΔSSI **(E)**.

**FIGURE 4:**
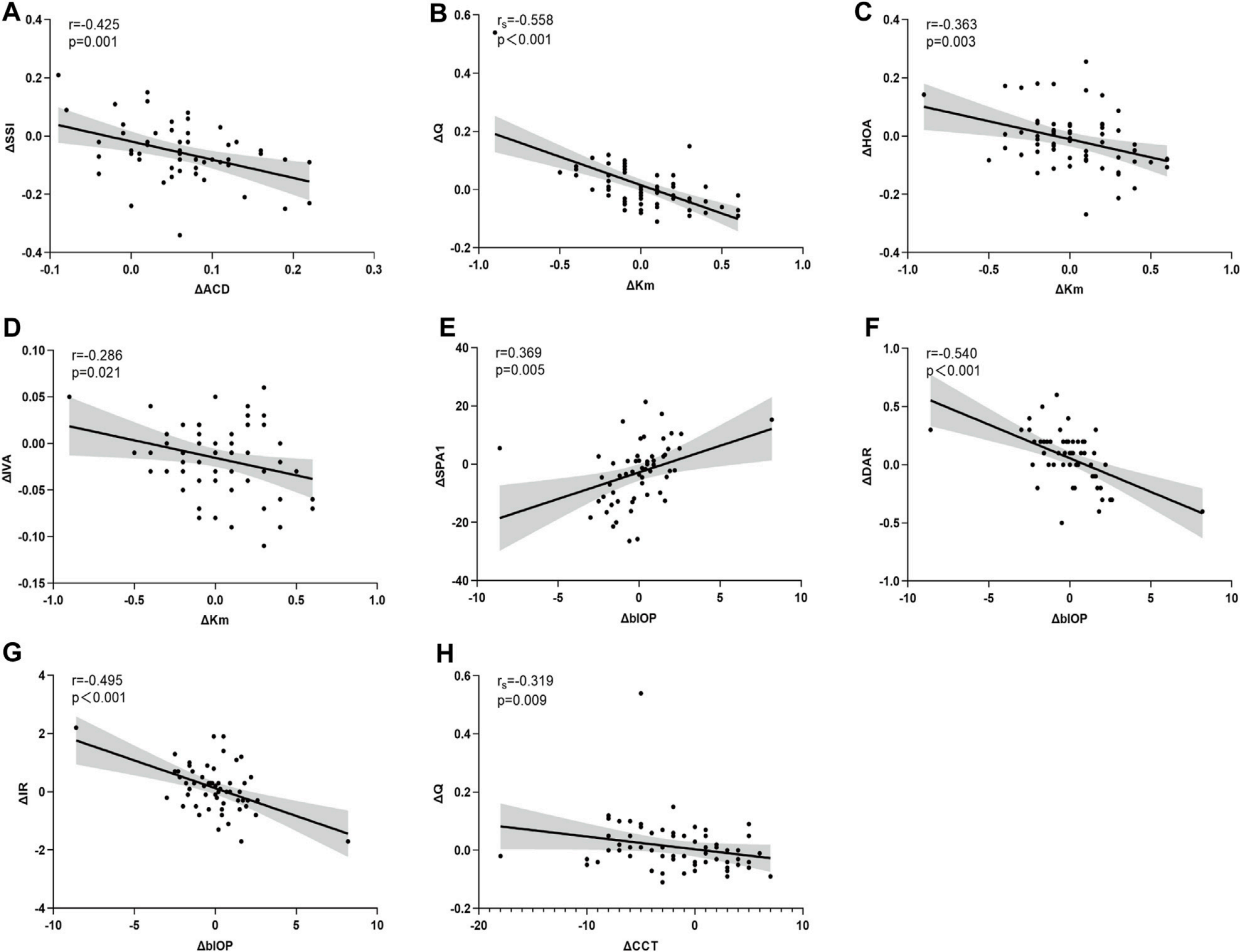
Asymmetry in corneal parameters are significantly correlated with asymmetry in ocular biometric parameters (Panel 4 A-H). ΔACD is significantly correlated with ΔSSI **(A)**. ΔKm is significantly correlated with ΔQ **(B)**, ΔHOA **(C)**, ΔIVA **(D)**. ΔblOP is significantly correlated with ΔSPA1 **(E)**, ΔDAR **(F)**, ΔIR **(G)**. ΔCCT is significantly correlated with ΔQ **(H)**.

Statistically significant correlations were identified between ΔSE and ΔIVA (r = 0.255, *p* = 0.040), ΔBAD-D (r = 0.360, *p* = 0.006), ΔSSI (r = 0.276, *p* = 0.039), and ΔDAR (r = −0.329, *p* = 0.013). Additionally, ΔAL demonstrated a significant correlation with ΔSSI (r = −0.319, *p* = 0.016) (Figures 3A–E). Notably, Δ mean keratometry (Km) and ΔbIOP were significantly correlated with specific corneal morphological and biomechanical parameters, respectively. Correlations involving other corneal and ocular parameters are detailed in Table 2 (Figures 4A–H).

**TABLE 2:** Correlation of asymmetry between corneal parameters and ocular biometric parameters.

Parameters	ΔSE(D)		ΔAL (mm)		ΔACD (mm)		ΔKm(D)		ΔbIOP (mmHg)		ΔCCT (μm)	
	*r*_*(s)*_	*p*	*r*_*(s)*_	*p*	*r*_*(s)*_	*p*	*r*_*(s)*_	*p*	*r*_*(s)*_	*p*	*r*_*(s)*_	*p*
Corneal morphological parameters												
ΔQ	−0.022	0.863	0.119	0.343	0.238	0.057	**−0.558**	**<0.001**	0.044	0.745	**−0.319**	**0.009**
ΔPCE	0.192	0.126	−0.126	0.318	0.092	0.465	−0.161	0.199	0.117	0.389	−0.066	0.603
ΔTOA	−0.064	0.614	0.065	0.607	0.130	0.302	0.094	0.457	−0.088	0.517	−0.036	0.775
ΔHOA	0.128	0.311	−0.170	0.176	0.112	0.375	**−0.363**	**0.003**	0.026	0.849	−0.223	0.074
ΔISV	0.126	0.317	−0.082	0.516	0.019	0.877	0.067	0.597	0.159	0.243	0.043	0.732
ΔIVA	**0.255**	**0.040**	−0.176	0.161	0.094	0.456	**−0.286**	**0.021**	0.132	0.332	0.059	0.640
ΔIHA	−0.141	0.264	0.192	0.125	0.064	0.614	−0.055	0.662	−0.174	0.199	0.215	0.086
ΔI-S	0.141	0.301	−0.028	0.838	0.178	0.188	0.012	0.927	0.005	0.969	0.007	0.957
ΔBAD-D	**0.360**	**0.006**	−0.255	0.058	0.085	0.534	0.114	0.403	−0.095	0.484	−0.032	0.817
Corneal biomechanical parameters												
ΔSSI	**0.276**	**0.039**	**−0.319**	**0.016**	**−0.425**	**0.001**	−0.020	0.884	−0.061	0.653	0.218	0.106
ΔSPA1	0.012	0.932	−0.043	0.753	−0.172	0.205	0.044	0.749	**0.369**	**0.005**	0.191	0.158
ΔARTh	−0.053	0.701	0.039	0.777	0.007	0.961	0.122	0.372	−0.143	0.294	0.050	0.716
ΔDAR	**−0.329**	**0.013**	0.249	0.065	0.066	0.627	0.234	0.082	**−0.540**	**<0.001**	−0.004	0.976
ΔIR	−0.189	0.163	0.227	0.093	0.249	0.065	0.054	0.693	**−0.495**	**<0.001**	−0.166	0.222
ΔCBI	−0.103	0.449	0.122	0.369	0.058	0.669	0.198	0.144	0.174	0.201	0.014	0.916
ΔTBI	0.231	0.086	−0.139	0.306	0.077	0.571	0.105	0.439	0.043	0.751	0.053	0.698

Bold values indicate *p* < 0.05.

### 3.4 Multiple regression analysis for predicting variations in myopic corneal parameters

The outcomes of the multiple regression analysis, as illustrated in Table 3, demonstrate that ΔSE exhibited a positive correlation with ΔIVA, ΔBAD-D, and ΔSSI and negatively correlated with ΔDAR (Standardized β coefficients were 0.260, 0.360, 0.226, and −0.284, respectively). Additionally, other ocular biometric parameters, such as ΔKm, ΔACD, and ΔbIOP, also affected certain corneal parameters with greater Standardized β coefficients than the ametropic parameters.

**TABLE 3:** Multiple regression analysis for predicting changes in myopic corneal parameters.

Parameters	Predictors	Unstandardized coefficient B	Standardized coefficient *β*	*p*-value	Adjusted R^2^
ΔSE	Constant	−0.473		<0.001	0.912
	ΔAL	−1.977	−0.957	<0.001	
	ΔLT	−1.955	−0.116	0.003	
ΔAL	Constant	<0.001		0.778	1.000
	ΔVL	1.001	0.989	<0.001	
	ΔLT	0.991	0.121	<0.001	
	ΔACD	0.969	0.117	<0.001	
ΔQ	Constant	0.016		0.073	0.372
	ΔKm	−0.195	−0.618	<0.001	
ΔIVA	Constant	0.009		0.452	0.122
	ΔKm	−0.038	−0.290	0.016	
	ΔSE	0.009	0.260	0.030	
ΔBAD-D	Constant	0.192		0.060	0.113
	ΔSE	0.094	0.360	0.006	
ΔSSI	Constant	0.035		0.290	0.202
	ΔACD	−0.587	−0.397	0.002	
	ΔSE	0.020	0.226	0.068	
ΔSPA1	Constant	−2.796		0.038	0.120
	ΔbIOP	1.825	0.369	0.005	
ΔDAR	Constant	0.064		0.097	0.391
	ΔbIOP	−0.054	−0.509	<0.001	
	ΔSE	−0.057	−0.284	0.010	
	ΔKm	0.213	0.241	0.026	
ΔIR	Constant	−0.045		0.719	0.274
	ΔbIOP	−0.188	−0.489	<0.001	
	ΔACD	2.855	0.235	0.046	

## 4 Discussion

The primary objective of this study is to explore the impact of myopia on a spectrum of corneal parameters. Prior investigations across diverse populations have indicated potential impairments in the corneal mechanical strength of myopia (Han et al., 2020; Liu et al., 2021; Liu Y. et al., 2022), alongside variances in ocular biometric traits (Niu et al., 2023). However, inherent individual variations pose challenges to research outcomes. This study employs a novel approach by utilizing a binocular comparison model, contrasting emmetropic and myopic eyes within cases of simple myopic anisometropia. This methodology ensures uniformity in genetic, environmental, and hormonal influences across both eyes, which have been shown to affect the incidence and progression of myopia. (Kunceviciene et al., 2019; Leshno et al., 2020; Luong et al., 2020; Van Mazijk et al., 2022). Thereby diminishing the impact of extraneous variables. Furthermore, anisometropia in this context serves as a metric for the extent of myopic alteration in emmetropic eyes. The association between anisometropia and corneal parameter asymmetry can be construed as a reflection of the interplay between myopia progression and the magnitude of corneal parameter changes. The stratification of anisometropia in our study is fundamentally grounded on the severity of myopia, facilitating a comparative analysis between low to moderate myopic and emmetropic states. Additionally, considering that astigmatism typically exhibits negligible alterations during normal myopic progression, our study excluded patients with astigmatic anisometropia to minimize astigmatism’s influence on the research findings.

The comparative analysis of ocular biological parameters indicates that the disparity in binocular AL was mainly from VL, aligning with findings by Hassan et al. (Hashemi et al., 2013). The main factors affecting the refractive status of the eye are corneal curvature and AL. Our study found there is a strong correlation between ΔAL and ΔSE, while the change of corneal curvature has little effect on refraction. Therefore, we hypothesise that anisometropia is mainly related to the asymmetry of AL, similar to Kinori’s conjecture. After analysing a decade-spanning and population-based dataset, Kinori et al. concluded that myopic anisometropia may reflect a difference in the rate of eye growth (AL) between the two eyes (Kinori et al., 2024). Relative to emmetropia, myopia exhibits an increased ACD and reduced LT, with these differences being minor yet statistically significant. Notably, significant variances are observed in corneal biological parameters between anisometropic eyes. Myopic eyes are characterized by thinner corneas, increased Kf, and reduced Ka, though no significant differences were noted in steep keratometry (Ks) and Km. These findings are congruent with those reported by Liu et al. (Liu Y. et al., 2022) in patients with varying degrees of myopia. Similarly, Gao et al. (Gao et al., 2022) observed reduced astigmatism and thinner corneas in relatively myopic eyes when comparing contralateral eyes in anisometropia, corroborating our results. However, their study revealed significant binocular differences in Km and bIOP, diverging from our findings. Wu et al. (Wu et al., 2019) also reported elevated IOP in high myopia in a disparate cohort study. We speculate that this may be related to the differences in subjects. In Gao’s study, the SE of relative myopic eyes was −3.74 ± 2.28D, significantly higher than the −2.93 ± 1.09D in our study. Moreover, Gao’s research subjects only required anisometropia, which may include many types, but our study only included patients with simple myopic anisometropia. The comparison data between low myopia and high myopia by Wu were from different individuals and was not a contralateral control for the same patient, which may be the reason for the significant difference with us. In addition, the number of participants in our study is relatively small, and sampling errors may also be one of the reasons for the differences. There are also studies that have yielded the same results as ours, for example, Sedaghat et al. (Sedaghat et al., 2021) showed no significant differences in Km and bIOP across varying degrees of myopia. And the conclusion of this section is still controversial.

In the realm of corneal morphometrics, the TOA in myopic corneas is found to be less than those in emmetropia, while no significant differences in high-order aberrations (6 mm) (HOA) are observed. This observation aligns with the findings of Sun et al. (Sun et al., 2022), who also reported reduced coma aberrations in more myopic eyes compared to their contralateral counterparts in anisometropic patients. The ISV quantifies the deviation of corneal curvature from the mean, and the IVA assesses the corneal curvature’s symmetry along the horizontal meridian. Notably, both ISV and IVA are reduced in myopia, suggesting a relative regularity in corneal symmetry (Shetty et al., 2017). This regularity may be associated with smaller corneal astigmatism in myopia, as evidenced by a correlation between Ka and both TOA and ISV in myopic eyes, though such a relationship is not observed with IVA. The BAD-D is a composite index that amalgamates corneal thickness and curvature data to provide a comprehensive assessment of corneal morphology (Hashemi et al., 2016). BAD-D and IVA are recommended as the most effective indicators for detecting subclinical keratoconus (Hashemi et al., 2016; Eliasy et al., 2019). In our multiple regression analysis, a positive correlation between ΔIVA and ΔBAD-D with ΔSE is identified, suggesting that corneal surface morphology becomes more symmetrical and regular in the process of myopia. To our knowledge, we are the first study to discover that the corneal surface morphology of myopia is relatively more symmetrical and regular in patients with anisometropia.

Within the scope of corneal biomechanical parameters, the SSI serves as a measure of corneal stiffness, founded on finite element modeling. It uniquely estimates corneal stiffness independently of bIOP and CCT (Eliasy et al., 2019), thus offering enhanced insight into corneal biomechanics (Chong and Dupps, 2021). The SPA1 is considered pivotal in evaluating corneal stiffness (Zhang et al., 2018). Observations indicate that both SSI and SPA1 are lower in myopic eyes compared to emmetropic counterparts, suggesting a reduction in corneal stiffness associated with myopia (Zhang et al., 2018; Eliasy et al., 2019; Chong and Dupps, 2021). Notably, ΔTBI is less pronounced in the high anisometropia group than in the low group. TBI represents a comprehensive amalgamation of tomographic topography and biomechanical indices, demonstrating high diagnostic efficacy for subclinical keratoconus detection. No significant difference was found in the Corvis Biomechanical Index (ΔCBI) between the two groups, and it is conjectured that ΔTBI outcomes may be influenced by the ΔBAD-D. The DAR relates to the magnitude of corneal deformation, quantifying the extent of material deformation under a specific load (Jędzierowska and Koprowski, 2019). Multiple regression analysis revealed that ΔSSI positively correlates with ΔSE, while ΔDAR inversely relates to ΔSE. These results echo the findings of Long et al. (Long et al., 2019) and Liu et al. (Liu Y. et al., 2022) in varied cohorts. However, Gao et al. (Gao et al., 2022) did not establish a definitive linear relationship between anisometropia and biomechanical asymmetry, possibly due to the inclusion of patients with compound myopic refractive parametrization in their study. We hypothesise that it may be due to the fact that Gao’s patient had myopic changes in the both corneas and thus the difference between the eyes was insignificant. Our findings suggest that with increasing myopia severity, there is a greater decrease in corneal stiffness, thereby rendering the cornea more prone to deformation. This conclusion aligns with existing research in the field (Yu et al., 2020; Liu et al., 2021; Ma et al., 2021; Liu Y. et al., 2022).

The findings of this study suggest a significant interrelation between the progression of myopia and alterations in corneal parameters. This notion is supported by the work of Ren et al. (Ren et al., 2023), who postulated and subsequently validated a similar hypothesis. They developed an AL increment model, based on a mathematical equation formulated by Morgan (Morgan et al., 2020). Their results indicated that the increment in AL might be a key factor influencing the corneal changes. Further exploring the possible causes, it was experimentally demonstrated that the elongation of AL associated with myopic progression is intricately linked to biochemical and ultrastructural modifications in the sclera. Given that the cornea and sclera originate from the same mesodermal layer and constitute the outer wall of the eyeball, it is plausible that similar changes occur in the cornea (Markov et al., 2018). During the process of myopia, the collagen fibers of the cornea gradually stretch and change the corneal geometry. In our study, the increase in ACD and changes in corneal curvature in myopic eyes can be used as auxiliary evidence, and some research reports indicate that the cornea has a tendency to become flatter with increasing myopia (Jonas et al., 2016; Kato et al., 2019). The morphological changes of the corneal surface may be due to the stretching of collagen fibers, which results in a straighter fibre alignment and thus a reduction in the “rugged terrain” of the fibrous layer. The morphology of the fibrous layer affects the smoothness and regularity of the corneal surface, which is partially reflected in the significant reduction of ISV and IVA in myopia. Therefore, we speculate that the relatively symmetrical and regular morphology of the corneal surface during myopia is a macroscopic manifestation of the altered morphology in collagen fibers. On the other hand, collagen fibers are the structural foundation of the cornea as a biological soft tissue, and the reduction in corneal biomechanics may be related to the remodeling of corneal collagen fibers during myopia (Stepp and Menko, 2021; Yu et al., 2024). In our study, the corneal thickness of myopia was significantly smaller. The diameter, quantity, proportion, and arrangement of collagen fibers may all change (Gao et al., 2022), which in turn affects the viscoelasticity of the corneal tissue and alters its stiffness and deformability. We hypothesise that this may explain the decrease in corneal stiffness in myopia. Recent studies have identified modifications in corneal biochemistry and ultrastructure in myopic eyes (Chen et al., 2021; Xin et al., 2021; Ni et al., 2023; Yu et al., 2024), including cellular transcription, proteomic analyses, and metabolomic analyses, which partially confirms our hypothesis. However, further research is needed on the specific changes in corneal collagen fibers and their relationship with corneal parameters.

While our study adopts a cross-sectional design, it poses limitations in deducing the temporal sequence and causal relationship between observed phenomena. There exists evidence suggesting that AL undergoes rapid elongation, particularly in the period before the onset of myopia (Xiang et al., 2012). It is plausible that changes in the composition or structure of both the cornea and sclera precede the development of myopia. In an exploratory study involving children aged four to six, Long et al. (Long et al., 2019) identified that a softer and more deformable cornea is a characteristic observed in childhood myopia. This leads to speculation on whether the more pliable corneal traits in emmetropic or hyperopic children could foretell the future onset of myopia. Therefore, monitoring corneal parameters could potentially serve as an early indicator for identifying children at risk of developing myopia. The exploration of the relationship between myopia and the extent of change in corneal parameters could provide valuable insights for predicting the future progression of myopia (Wu et al., 2019; Bataille et al., 2021), which would be instrumental in myopia prevention and control strategies. Future research endeavors should consider the potential of corneal parameters in predicting the development of myopia in children, providing early warning for corneal parameter values in children who are likely to be myopic and guiding clinical interventions to prevent myopia as soon as possible.

In the context of a predictive model linking myopia with corneal parameters, it has been posited (Sedaghat et al., 2021) that among various factors, the deformability parameter, reflecting the elastic properties of corneal collagen fibers, is crucial. Specifically, the corneal curvature radius at the highest concavity (HCR) demonstrates the most substantial correlation with scleral influence and emerges as the predominant predictor of myopic severity. Additional research suggests that the peak distance (PD) serves as a notable marker for high myopia (Ma et al., 2021). In our investigation, BAD-D and DAR both emerge as effective predictors. It is important to note that AL elongation tends to be relatively uniform during the physiological progression of myopia. However, in cases of pathological myopia, the extension is primarily concentrated at the posterior pole, with anterior structural changes remaining relatively stable (Liu J. et al., 2022). Consequently, the correlation with AL diminishes (Liu et al., 2021; Sedaghat et al., 2021). Liu et al. (Liu J. et al., 2022) observed no significant differences in ACD, CCT, Km, and LT among highly myopic eyes with anisometropia. Furthermore, Liu et al. (Liu et al., 2021) reported a lack of significant correlation between SSI and AL in eyes with severe axial elongation (AL≥26 mm), suggesting a non-linear influence of AL on corneal parameters. SSI does not decrease with increasing myopia, but gradually stabilizes at a lower level. Similarly, our study showed a positive correlation between ΔSE and ΔSSI, but there was no statistically significant difference in ΔSSI across low and high anisometropic groups. This may be potentially due to uneven growth of the eyeball. This may restrict the applicability of corneal parameter predictions, indicating a need for further exploration.

The current study has some limitations that warrant consideration when interpreting its findings. Primarily, its cross-sectional design inherently restricts the capacity to capture dynamic characteristics and establish causal relationships among the variables examined. Therefore, prospective studies are needed to elucidate the underlying mechanisms of the onset and advancement of myopia in children with anisometropia. Additionally, the relatively small sample size of this investigation might introduce potential biases in the outcomes. Due to the limitation of anisometropia, the patients only have low to moderate myopia and exclude higher myopia. Notably, corneal changes have a non-linear relationship with myopia, particularly in the context of pathological myopia, posing challenges to accurate prediction. This may affect the broader applicability of our research results.

In Conclusion, myopic eyes have thinner corneas and smaller corneal astigmatism, and exhibit corneal morphologies that are relatively more symmetrical and regular compared to contralateral emmetropia in anisometropia patients. However, myopic corneas tend to be more susceptible to deformation and possess somewhat compromised biomechanical properties. In addition, a discernible linear relationship is evident between anisometropia and asymmetry of corneal parameters, which further suggests a correlation between increased myopia and alterations in corneal parameters. This association could potentially serve as a predictive marker of myopia and guide clinical interventions to prevent myopia. Future studies, ideally on a larger scale, should be conducted to substantiate these findings and further explore this predictive potential.

## Data Availability

The original contributions presented in the study are included in the article/Supplementary Material, further inquiries can be directed to the corresponding author.
